# Prevalence of and Factors Associated with Rectal-Only Chlamydia and Gonorrhoea in Women and in Men Who Have Sex with Men

**DOI:** 10.1371/journal.pone.0140297

**Published:** 2015-10-29

**Authors:** Geneviève A. F. S. van Liere, Martijn S. van Rooijen, Christian J. P. A. Hoebe, Titia Heijman, Henry J. C. de Vries, Nicole H. T. M. Dukers-Muijrers

**Affiliations:** 1 Department of Sexual Health, Infectious Diseases and Environmental Health, South Limburg Public Health Service, Geleen, The Netherlands; 2 Department of Medical Microbiology, School of Public Health and Primary Care (CAPHRI), Maastricht University Medical Center (MUMC+), Maastricht, The Netherlands; 3 Public Health Laboratory, Public Health Service of Amsterdam (GGD Amsterdam), Amsterdam, The Netherlands; 4 Department of Research, Public Health Service of Amsterdam (GGD Amsterdam), Amsterdam, The Netherlands; 5 STI Outpatient Clinic, Public Health Service of Amsterdam (GGD Amsterdam), Amsterdam, The Netherlands; 6 Department of Dermatology, Academic Medical Center (AMC), University of Amsterdam, Amsterdam, The Netherlands; 7 Centre for Infection and Immunology Amsterdam (CINIMA), Academic Medical Center (AMC), Amsterdam, The Netherlands; David Geffen School of Medicine at UCLA, UNITED STATES

## Abstract

**Background:**

Both anorectal *Chlamydia trachomatis* (CT) and *Neisseria gonorrhoea* (NG) can occur as a rectal-only infection or concurrently with simultaneous urogenital infection with the same pathogen. Characterising the target groups in which rectal-only infections occur may improve the efficacy of screening practices.

**Methods:**

We analysed data from two Dutch outpatient sexually transmitted infection (STI) clinics between 2011 and 2012. We included all men who have sex with men (MSM) (n = 9549) and women (n = 11113), ≥18 years, who had been tested for anorectal and urogenital CT and/or NG (either as a result of reporting anal sex/symptoms or via routine universal testing). Factors associated with rectal-only CT and NG infections were assessed using univariable and multivariable logistic regression.

**Results:**

In MSM, anorectal CT prevalence was 9.8% (693/7094), anorectal NG prevalence was 4.2% (397/9534). In women this was 9.5% overall (439/4597) and 0.9% (96/10972) respectively. Anorectal CT prevalence among women who were routinely universally tested was 10.4% (20/192), for selective testing this was 9.5% (419/4405) (p = 0.68). Anorectal NG infections were not detected among women who were routinely universally tested (p = 0.19). Among CT or NG positive MSM, rectal-only CT infections were found in 85.9% (595/693), for NG this was 85.6% (340/397) respectively. In positive women these figures were 22.1% (97/439)for CT and 20.8% (20/96) for NG, respectively. In MSM, independent factors associated with rectal-only CT were: being a sex worker (OR0.4,CI0.2–1.0), exclusively having sex with men (OR3.4,CI1.7–6.8), and absence of urogenital symptoms (OR0.2,CI0.2–0.4). In women, these factors were: older age (OR2.3, CI1.3–4.0) and non-Western nationality (OR1.8, CI1.0–3.5). Factors associated with rectal-only NG in MSM were: having been warned for STIs by an (ex) partner (OR2.9,CI1.1–7.5), oropharyngeal NG infection (OR2.4,CI1.0–5.3), and absence of urogenital symptoms (OR0.02,CI0.01–0.04), while in women no significant factors were identified.

**Conclusions:**

The prevalence of anorectal CT and NG was substantial in MSM and prevalence of anorectal CT was also substantial in women. Anorectal infections occurred mostly as rectal-only infections in MSM and mostly concurrent with other infections in women. Given the lack of useful indicators for rectal-only infections, selective screening based on a priori patient characteristics will have low discriminatory power both in relation to MSM and women.

## Introduction

With the introduction of sensitive nucleic acid amplification assays (NAAT) tests, anorectal testing for *Chlamydia trachomatis* (CT) and *Neisseria gonorrhoea* (NG) has become more commonplace in sexually transmitted infection (STI) clinics[1,2]. Anorectal CT and NG are common in men who have sex with men (MSM) and in women[3]. Anorectal testing is important since the majority of infections are asymptomatic. Undetected infections could lead to further spread of infection in the population and the development of sequelae within individuals. Moreover, anorectal CT and NG infections facilitate HIV transmission[4].

There is a lack of international consensus regarding the adequate treatment for anorectal CT. Guidelines in the US recommend single-dose azithromycin or a 7-day course of doxycycline as equally effective treatments for uncomplicated anorectal CT in MSM and non-pregnant women[5]. In the Netherlands and the UK, doxycycline is recommended for anorectal CT[6,7].

Prevalence of anorectal CT has been reported to be as high as 24.4%[3,8–17] among MSM and 17.5% among women[3,13–15,18–25], with rates for NG as high as 17.9%[3,8–11,13–15,17] and 13.4%[3,11,13–15,18,19,22–25]for MSM and women respectively. Insight into the factors associated with anorectal infections, including demographic and behavioural factors, can facilitate the identification of high-risk groups and inform guidelines on anorectal testing. Previous studies in MSM and women attending STI clinics have shown that being of a younger age[9,11] and having multiple sex partners[8,11] are both associated with having anorectal CT and NG infections. Notably, anal sex has not been found to be associated with anorectal CT, but has been associated with anorectal NG[13,14].

Anorectal infections can be rectal-only infections, i.e., infection at the anorectal site only, or can occur concurrently with simultaneous urogenital and/or oropharyngeal infections with the same pathogen. Previous studies in MSM have reported a high proportion of rectal-only CT (up to 90%) and NG infections (up to 70%)[8–11]. In contrast, in women, a relatively low proportion of rectal-only CT and NG infections have been reported (between 0% to 44%)[18,19,22–27]. It is unknown which factors determine such differences in the relative proportions of rectal-only infections. These differences may be related to e.g. sexual risk behaviour or anatomical differences, but are probably not caused by the characteristics of the CT strain[28]. If a single dose of azithromycin is effective in treating anorectal CT, concurrent urogenital and anorectal CT infections would also be opportunistically treated[13], as is the case with concurrent NG infections. However, rectal-only CT and NG infections would not be opportunistically treated with a urogenital infection. Treatment of rectal-only infections depends on screening algorithms, as they are not routinely universally tested for in practice. Insight into the proportion of rectal-only infections and the factors associated with them could help to define individuals at risk for such untreated anorectal infection. This is important for the design of cost-effective screening guidelines specifying who should be tested at which anatomical sites.

By evaluating 20.662 unique STI clinic attendees tested at both urogenital and anorectal sites, this study aims to increase understanding of anorectal infections by assessing the prevalence of and factors associated with (rectal-only) anorectal CT and NG in MSM and in women.

## Methods

### Study Population

**Fig 1:**
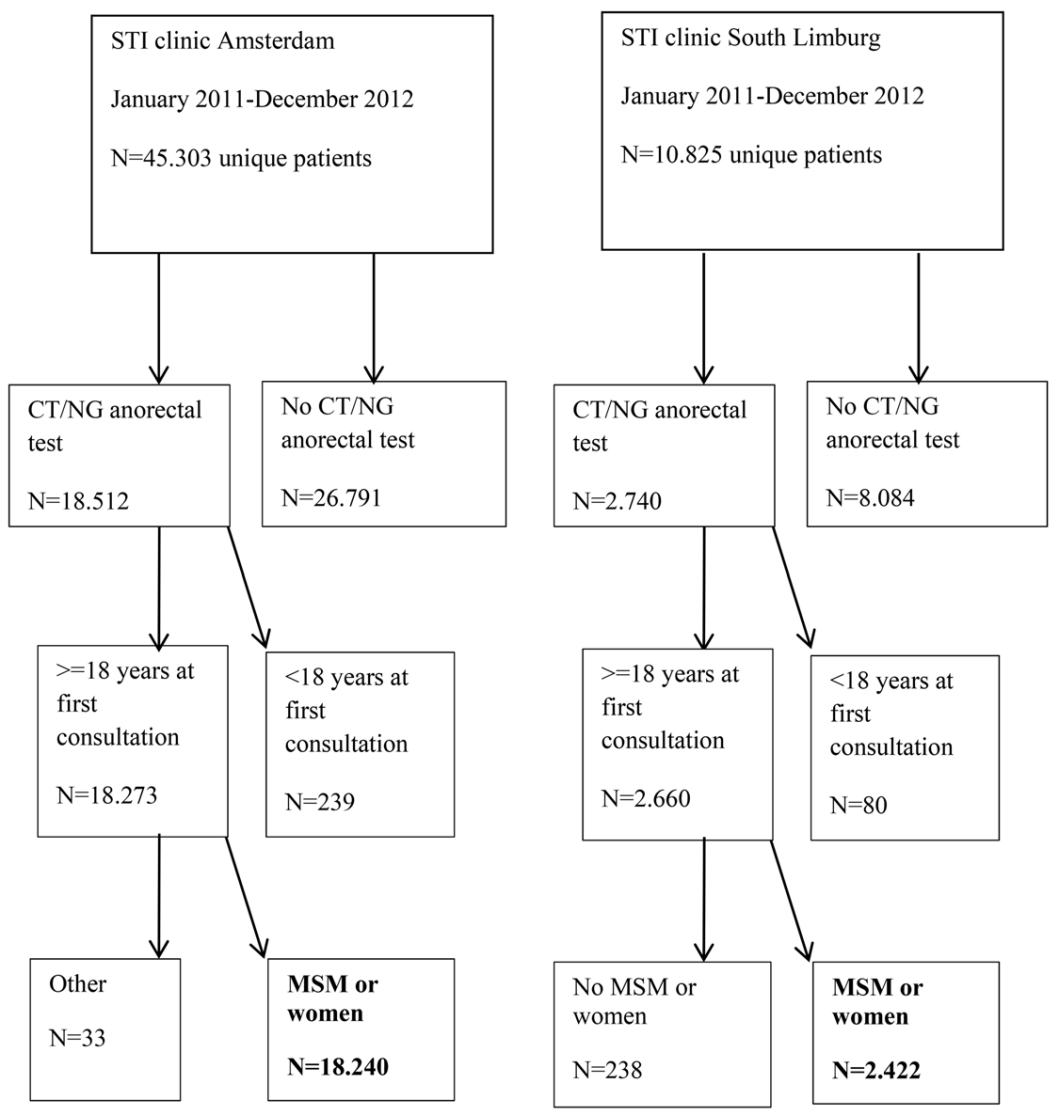
Flowchart of the study population.

This study was approved by the Medical Ethical Committee of the University of Maastricht (METC 11-4-108), who waived the need for consent to be collected from participants. Since retrospective data originated from standard care (in which one can opt-out for the use of their data for scientific research, as approved by METC 11-4-108) and were analysed anonymously, no further informed consent for data analysis was obtained. The outpatient Public Health Service STI clinics in Amsterdam (approximately 36.000 consultations in 2012) and South Limburg (approximately 6500 consultations in 2012) offer free and anonymous STI testing to high-risk individuals. From January 2011 to December 2012, data from all MSM and women who had been tested for both urogenital and anorectal CT and/or NG were included once, from their most recent consultation (N = 20.662 unique individuals, 9549 MSM and 11113 women) (Fig 1). MSM were defined as men who had sex with men in the past 6 months.

### Study Procedures

All participants were routinely tested for urogenital CT and NG infections via a urine or urethral sample (men) or a vaginal or cervical swab (women).

In Amsterdam, if receptive anal sex was reported in the past 6 months, both MSM and women were screened for anorectal CT and NG infections. Irrespective of reported anal sex, MSM and high-risk women were routinely screened for anorectal NG infection. High-risk women who report active oral sex in the previous 6 months (regardless of condom use) and all MSM (irrespective of reported receptive oral sex) were routinely tested for oropharyngeal CT and NG. High-risk women were defined as: women who report symptoms, women who are commercial sex workers (CSW), women who have been warned for STIs by an (ex-) partner, or women who have been referred by another healthcare provider.

In South Limburg, for study purposes, between May and December 2012 women attending one of the 3 (out of 13) study nurses were routinely universally screened for anorectal CT and NG; this comprised 16% (192/1200) of women tested at anorectal site in South Limburg[20]. Before and after this period, women were only tested at the anorectal site if they reported having anal sex or having anal symptoms. All MSM were routinely screened for anorectal CT and NG, irrespective of reported behaviour.

If receptive oral sex was reported to have taken place in the past 6 months, swingers and CSW were tested for oropharyngeal CT and NG infections. Other women were tested if there was a risk for an oropharyngeal-only infection, that is, without concurrent urogenital or anorectal infection, in addition to reported receptive oral sex. MSM were routinely universally screened for oropharyngeal CT and NG infections.

Each consultation included a standardised medical and sexual history taken by nurses, including self-reported symptoms and sexual behaviour over the past six months. Specimens tested consisted of vaginal/cervical swabs or urine, anorectal swabs and oropharyngeal swabs, either self-collected or collected by the nurse. All tests were performed according to the manufacturer’s protocol. In the South Limburg clinic, specimens were processed at two regional laboratories using three different NAATs (SDA, Becton Dickinson ProbeTec ET system, Maryland, USA; Cobas Amplicor, Roche, California, USA; Cobas 4800, Roche, California, USA). In the Amsterdam clinic, the Aptima combo CT/NG assay for the detection of *C*. *trachomatis* and *N*. *gonorrhoeae* rRNA (Hologic Gen-Probe Inc., San Diego, USA) and the Aptima Ct assay were used. In Amsterdam, NG was routinely tested in MSM and high-risk women by culture. Serum was tested for Treponema pallidum haemagglutination (TPHA) (Bioelisa Syphilis 3.0 (Biokit, SA, USA)) and HIV (anti-HIV1/2, Axsym; Abbott Laboratories, Chicago, IL, USA). Reactive samples were confirmed using Western blot (South Limburg; HIVblot 2.2; Genelabs Diagnostics, Science Park, Singapore, Amsterdam; INNO-LIA HIV I/II Score; Fujirebio; 201 Great Valley Parkway, Malvern, USA).

### Statistical Analyses

The prevalence of anorectal CT and NG was calculated by dividing the number of positive tests by the total number of tests, multiplied by 100. Anorectal CT prevalence is reported separately for women who were routinely universally tested and women who were tested based on the report of anal sex and/or symptoms (selective testing). Anorectal NG infections were reported overall, since anorectal NG was detected among women who were routinely universally tested (p = 0.19). Rectal-only infections were defined as an anorectal CT or NG infection without a concurrent urogenital infection with the same pathogen. Concurrent infections were defined as simultaneous urogenital and anorectal infection with the same pathogen. Oropharyngeal CT or NG infections were not taken into account in this classification, but assessed as a possible associated factor and presented in the tables. In all individuals, univariable and multivariable logistic regression were used to identify factors (independently) associated with (1) anorectal CT and anorectal NG and (2) among anorectal CT or NG positive individuals only, with rectal-only CT and NG infections compared to concurrent infections. Several variables were examined based on self-reports of behaviour during the 6 month period prior to consultation. The variables tested were: age, nationality, CSW, sexual preference (for MSM: exclusively having sex with men or not), number of sex partners, antibiotic use in the past 1–3 months, sexual practices (anal/vaginal), condom use (anorectal/vaginal), intravenous (IV) drug use, having been warned for STIs by an (ex) partner (as in partner notification), symptoms (anorectal/urogenital), concurrent oropharyngeal CT, concurrent oropharyngeal NG, HIV status, TPHA positivity, and history of STI clinic consultations in the past 800 days. This last variable was divided into two variables: one for CT and one for NG. The CT variable was divided in the following mutually exclusive categories: (1) not tested, (2) previously tested CT negative, (3) previously had concurrent anorectal CT and (4) previously had at least one rectal-only CT. The NG variable was divided in the following mutually exclusive categories: (1) not tested, (2) previously tested NG negative, (3) previously had concurrent anorectal NG and (4) previously had at least one rectal-only NG. The variables age (for MSM ≤32, 33–43, ≥44, and for women ≤22, 23–27, ≥28), number of sex partners in the past 6 months (≤2, 3–5, ≥6), and number of previous STI clinic consultations (≤1, 2–3, ≥4), were all categorised into three groups based on tertile distributions. Urogenital symptoms were defined as: genital discharge, bleeding, itching, ulceration, swelling, pain, burning sensation and more frequent urination. Anal symptoms were defined as: anal discharge, bleeding, itching, ulceration, redness, swelling, pain and burning sensation. Anal sex was defined as insertive, receptive, or both. Anal condom use was categorised in the following way: ‘no anal sex’, ‘always’ and ‘not always’. A similar variable was constructed for vaginal condom use. The factors anal sex and anal symptoms were excluded from multivariable analyses exploring associations with anorectal CT positivity in order to prevent bias by testing indication (selective testing based on report of anal sex/symptoms). Variables with p < 0.05 were included with stepwise backward method in the multivariable model. In cases where variables were correlated (> 0.6), the variable with the lowest p-value in univariable analyses was omitted from the multivariable model. All multivariable models were corrected for study site (Amsterdam/South Limburg). The results of the univariable analyses are included in the supplemental files. The results of the multivariable analyses are included in the manuscript. A p-value < 0.05 was considered statistically significant. Analyses were performed using SPSS version 19.0 (IBM Inc., Somers, NY).

We visualised which factors would yield the highest number of infections if targeted in screening (i.e. if used as a screening indicator). Factors were selected based on their statistical significance in unadjusted univariable analyses and their usability as a screening tool, by including factors that were available from the patient at screening (i.e., excluding any test result from that screening).

The proportion of anorectal CT and NG infections diagnosed, i.e. the number of diagnoses within a category divided by the total number of diagnoses multiplied by 100, is presented in a bubble in order to visualise the yield of anorectal infections per factor. Thereby, the bubble represents the relative share in percentage of anorectal CT and NG infections detected. Each bubble represents an associated factor, and individuals can appear in multiple bubbles. The number of factors presented in one bubble plot was maximized to 8, to ensure clarity of the figure.

## Results

In the study period, 45303 unique individuals visited the STI clinic in Amsterdam, for South Limburg this was 10995 (Fig 1). In total, 20662 unique study participants who were tested for anorectal CT and/or NG (18240 Amsterdam, 2422 South Limburg) were included in our study; 9549 MSM and 11113 women. In total, 99.2% (n = 20506) were tested for NG and 56.6% (n = 11691) were tested for CT. The median age of MSM was 37 years (IQR 28–46), and the median age of women was 25 years (IQR 22–30). Anal sex was reported by 83.0% (n = 7921) of MSM and by 33.3% (n = 3696) of women, and anal symptoms were reported by 4.9% (n = 449) of MSM and 1.9% (n = 210) of women (Tables 1 and 2).

**Table 1:** Prevalence and factors associated with anorectal chlamydia and gonorrhoea and prevalence and factors associated with rectal-only chlamydia and gonorrhoea in men who have sex with men by multivariable logistic regression. Adjusted odds ratios (aORs) were adjusted for the variables included in this table. For categorical variables, the reference category is indicated with value ‘1’.

		Chlamydia								Gonorrhoea							
	Total MSM	Anorectal CT prevalence				Rectal-only CT				Anorectal NG prevalence				Rectal-only CT			
	% (n)	% (n) positive	aOR	p value	95% CI	% (n)	aOR	p value	95% CI	% (n) positive	aOR	p value	95% CI	% (n)	aOR	p value	95% CI
Datasource																	
Amsterdam	87.2 (8327)	10.1 (593)	1			87.4 (518)	1			4.3 (356)	1			86.2 (307)	1		
South Limburg	12.8 (1222)	8.2 (100)	0.8	0.45	0.6–1.0	75.0 (75)	0.8	0.53	0.4–1.6	3.4 (41)	0.7	0.14	0.6–1.1	80.5 (33)	0.9	0.92	0.2–4.1
Age																	
≤32	37.8 (3612)	11.1 (298)	1			84.6 (252)	ns			5.3 (191)	1			85.3 (163)	ns		
33–43	30.4 (2901)	10.0 (224)	0.9	0.14	0.97–1.5	87.1 (195)	ns			4.0 (115)	0.7	0.32	0.6–0.97	86.1 (99)	ns		
≥44	31.8 (3036)	8.0 (171)	0.8	<0.0001	0.6–0.96	85.4 (146)	ns			3.0 (91)	0.6	<0.001	0.4–0.8	85.7 (78)	ns		
Transmission group																	
MSWM	86.7 (8283)	7.0 (53)	1			62.3 (33)	1			2.1 (26)	ns			73.1 (19)	ns		
MSM	13.3 (1266)	10.1 (640)	1.4	0.04	1.02–2.0	87.5 (560)	3.4	0.001	1.7–6.8	4.5 (371)	ns			86.5 (321)	ns		
CSW																	
No	97.3 (9290)	9.6 (660)	ns			86.5 (571)	1			4.2 (389)	ns			85.9 (334)	ns		
Yes	2.7 (259)	15.9 (33)	ns			66.7 (22)	0.4	0.04	0.2–0.95	3.1 (8)	ns			75.0 (6)	ns		
Number sex partners																	
1	24.8 (2365)	7.5 (128)	1			86.7 (111)	ns			2.5 (60)	1			90.0 (54)	ns		
2	32.3 (3085)	9.7 (214)	1.3	0.04	1.01–1.7	87.9 (188)	ns			3.6 (112)	1.2	0.31	0.9–1.7	83.0 (93)	ns		
3+	42.6 (4064)	11.0 (344)	1.5	0.001	1.2–1.9	84.3 (290)	ns			5.5 (222)	1.7	0.002	1.2–2.3	85.6 (190)	ns		
Antibiotics																	
No	85.7 (8186)	10.4 (618)	1			86.1(532)	ns			4.3 (350)	ns			86.0 (301)	ns		
Yes	10.4 (991)	5.9 (44)	0.5	<0.0001	0.3–0.6	86.4 (38)	ns			3.2 (32)	ns			84.4 (27)	ns		
Warned																	
No	80.8 (7716)	8.2 (461)	1			86.6 (399)	ns			3.0 (234)	1			80.3 (188)	1		
Yes	15.6 (1489)	18.5 (203)	2.0	<0.0001	1.6–2.4	84.2 (171)	ns			10.0 (149)	2.6	<0.0001	2.0–3.3	94.6 (141)	2.9	0.03	1.1–7.5
TPHA positive																	
No	2.4 (227)	8.8 (20)	1			85.0 (17)	ns			3.1 (7)	ns			100 (7)	ns		
Not tested	93.4 (8919)	9.3 (605)	1.0	0.69	0.6–1.7	85.6 (518)	ns			4.0 (360)	ns			85.3 (307)	ns		
Yes	4.2 (403)	20.0 (68)	2.2	<0.0001	1.6–3.1	85.3 (58)	ns			7.4 (30)	ns			86.7 (26)	ns		
HIV																	
No	79.1 (7557)	8.0 (433)	1			84.5 (366)	ns			3.2 (238)	1			84.0 (200)	ns		
Yes	2.7 (257)	23.1 (55)	2.3	<0.0001	1.6–3.4	83.6 (46)	ns			9.3 (24)	2.3	0.003	1.3–3.8	83.3 (20)	ns		
Unknown	18.2 (1735)	14.1 (205)	1.7	<0.0001	1.4–2.1	88.3 (181)	ns			7.8 (135)	2.1	<0.0001	1.6–2.8	88.9 (120)	ns		
Urogenital symptoms																	
No	81.3 (7763)	9.5 (558)	ns			89.4 (499)	1			3.8 (295)	ns			98.3 (290)	1		
Yes	15.1 (1442)	12.1 (106)	ns			67.0 (71)	0.2	<0.0001	0.2–0.4	6.1 (88)	ns			44.3 (39)	0.02	<0.001	0.01–0.04
Ct urogenital																	
No	95.4 (9109)	8.7 (593)	1			100 (572)	ns			4.0 (363)	ns			87.6 (318)	ns		
Yes	4.3 (412)	38.2 (100)	5.2	<0.0001	3.9–7.0	0.0 (0)	ns			8.3 (34)	ns			64.7 (22)	ns		
Ct oropharyngeal																	
No	1.2 (119)	6.5 (6)	1			83.3 (5)	ns			1.7 (2)	ns			50.0 (1)	ns		
Not tested	97.4 (9301)	9.3 (639)	1.0	0.98	0.4–2.4	86.1 (550)	ns			4.1 (385)	ns			86.0 (331)	ns		
Yes	1.4 (129)	46.2 (48)	7.3	<0.0001	4.7–11.3	79.2(38)	ns			7.8 (10)	ns			80.0 (8)	ns		
Ng urogenital																	
No	97.5 (9308)	9.7 (674)	na			86.2 (581)	ns			3.7 (40)	1			100 (340)	ns		
Yes	2.5 (234)	14.1 (19)	ns			63.2 (12)	ns			24.5 (57)	5.1	<0.0001	3.4–7.7	0 (0)	ns		
Ng oropharyngeal																	
No	0.5 (50)	4.2 (1)	ns			100 (1)	ns			2.0 (1)	1			89.2 (223)	1		
Not tested	94.4 (9017)	9.6 (638)	ns			85.9 (548)	ns			2.8 (250)	na		na	80.1 (117)	na		na
Yes	5.0 (482)	14.5 (54)	ns			81.5 (44)	ns			30.4 (146)	10.2	<0.0001	7.8–13.4	86.7 (176)	0.4		0.2–0.95
N previous tests																	
0	53.3 (5144)	10.9 (395)	1			86.3 (341)	ns			4.0 (203)	ns			84.7 (122)	ns		
1–2	28.2 (2690)	10.0 (198)	0.8	0.60	0.4–1.8	85.4 (169)	ns			4.5 (120)	ns			82.4 (28)			
3+	18.0 (1714)	6.8 (100)	0.4	0.02	0.2–0.9	83.0 (83)	ns			4.3 (74)	ns			87.5 (14)			
Previous anorectal CT testing																	
No Ct at inclusion	22.2 (2122)	na	na		na	na	ns			0.7 (15)	0.1	<0.0001	0.1–0.3	73.3 (11)	ns		
Never tested before	41.7 (3985)	10.5 (419)	0.7	0.30	0.4–1.3	85.2 (357)	ns			5.2 (207)	1.3	0.42	0.7–2.5	87.4 (181)	ns		
Tested negative	28.8 (2753)	7.4 (181)	1			86.7 (157)	ns			4.0 (111)	1			81.1 (90)	ns		
Concurrent infection	0.9 (87)	16.0 (12)	1.7	0.16	0.8–3.6	100.0 (12)	ns			3.4 (3)	0.5	0.24	0.1–1.7	100 (3)	ns		
Rectal-only infection	6.3 (602)	14.2 (81)	1.6	0.01	1.1–2.2	82.7 (67)	ns			10.1 (61)	1.6	0.02	1.1–2.5	90.2 (55)	ns		
Previous anorectal NG testing																	
Never tested before	56.2 (5363)	10.8 (405)	1.3	0.55	0.5–3.4	85.4 (346)	ns			3.9 (209)	1.0	0.97	0.5–1.8	86.1 (180)	ns		
Tested negative	39.2 (3741)	7.5 (218)	1			87.6 (191)	ns			3.7 (138)	1			85.5 (118)	ns		
Concurrent infection	0.6 (55)	14.9(7)	1.6	0.33	0.6–3.8	85.7 (6)	ns			14.5 (8)	2.7	0.05	1.01–7.1	75.0 (6)	ns		
Rectal-only infection	3.9 (376)	17.3 (61)	1.9	0.01	1.3–2.7	78.7 (48)	ns			11.2 (42)	1.8	0.01	1.2–2.9	85.7 (36)	ns		

**Table 2:** Prevalence and factors associated with anorectal chlamydia and gonorrhoea and prevalence and factors associated with rectal-only chlamydia and gonorrhoea in women by multivariable logistic regression. Adjusted odds ratios (aORs) were adjusted for the variables included in this table. For categorical variables, the reference category is indicated with value ‘1’.

				Chlamydia							Gonorrhoea						
	Total women	Anorectal CT prevalence				Rectal-only CT				Anorectal NG prevalence				Rectal only NG			
	% (n)	% (n) positive	aOR	p value	95% CI	% (n)	aOR	p value	95% CI	% (n) positive	aOR	p value	95% CI	% (n)	aOR	p value	95% CI
Datasource																	
Amsterdam	89.2 (9913)	10.0 (339)	1			24.8 (84)	1			0.9 (87)	1			20.7 (18)			
South Limburg	10.8 (1200)	8.3 (100)	0.4	<0.0001	0.3–0.6	13.0 (13)	0.5	0.01	0.2–0.9	0.8 (9)	0.9	0.2	0.4–1.7	22.2 (2)	1.1	0.91	0.2–5.7
Age																	
≤22	32.1 (3571)	14.7 (193)	1.7	0.01	1.1–2.5	17.1 (23)	1			1.5 (51)	ns			15.7 (8)	ns		
23–27	34.0 (3782)	9.3 (132)	0.7	0.16	0.5–1.1	20.5 (27)	1.1	0.62	0.6–2.0	0.6 (23)	ns			26.1 (6)	ns		
≥28	33.8 (3760)	6.1 (114)	1			32.5 (37)	2.3	0.001	1.3–4.0	0.6 (22)	ns			27.3 (6)	ns		
Nationality																	
Western	84.8 (9422)	9.7 (386)	ns			20.2 (78)	1			0.9 (81)	ns			21.0 (17)	ns		
Non Western	14.7 (1633)	9.2 (52)	ns			36.5 (19)	1.8	0.05	1.0–3.5	0.9 (14)	ns			14.3 (2)	ns		
Ct urogenital																	
No	88.2 (9801)	2.3 (97)	1			100.0 (97)	ns			0.6 (54)	ns			24.1 (13)	ns		
Yes	11.7 (1304)	79.2 (342)	190.1	<0.0001	133.3–271.0	0.0 (0)	ns			3.2 (42)	ns			16.7 (7)	ns		
Ct anorectal																	
Not tested	58.6 (6516)		ns				ns			0.7 (47)	0.5	0.02	0.3–0.9	19.1 (9)	ns		
No	37.4 (4158)		ns				ns			0.8 (32)	1			28.1 (9)	ns		
Yes	4.0 (439)		ns				ns			3.9 (17)	3.2	0.01	1.3–8.2	11.8 (2)	ns		
Ng urogenital																	
No	97.3 (10817)	9.5 (417)	ns			22.3 (93)	ns			0.2 (20)	1			100 (20)	ns		
Yes	1.5 (165)	29.5 (18)	ns			22.2 (4)	ns			46.3 (76)	508.1	<0.001	287.2–899.2	0 (0)	ns		
N previous tests																	
0	67.1 (7455)	9.5 (292)	3.1	0.003	1.5–6.5	20.9 (61)	ns			0.8 (58)	ns			15.5 (9)	ns		
1–2	26.4 (2937)	11.3 (130)	4.3	<0.0001	2.0–9.4	25.4 (33)	ns			1.1 (33)	ns			30.3 (10)	ns		
3+	6.5 (721)	4.6 (17)	1			17.6 (3)	ns			0.7 (5)	ns			20.0 (1)	ns		

### Anorectal CT and NG Prevalence

The prevalence of anorectal CT was 9.8% (693/7094) in MSM and 9.5% (439/4597) in women (*p* = 0.86). Anorectal CT prevalence among women who were routinely universally tested was 10.4% (20/192), for selective testing this was 9.5% (419/4405) (p = 0.68). The prevalence of anorectal NG in MSM was 4.2% (397/9534), in women this was 0.9% (96/10972) (*p* < 0.001).

### Prevalence of Rectal-Only Infections

**Fig 2:**
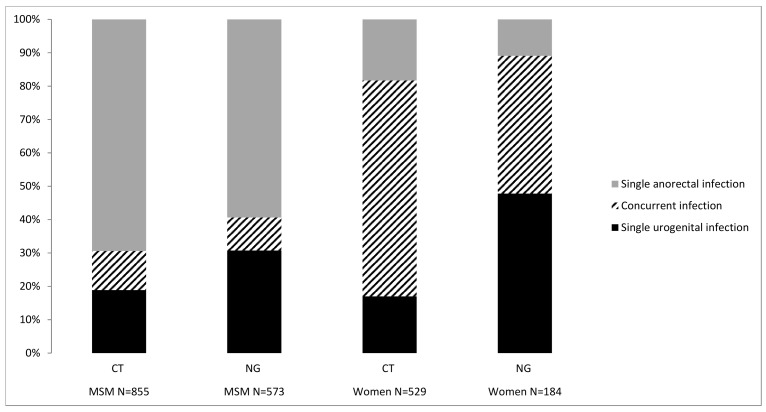
Anatomic site distribution of CT or NG positive MSM and women both tested at urogenital and anorectal sites.

In MSM, 162 urogenital and 693 anorectal CT infections and 176 urogenital and 397 anorectal NG infections were diagnosed. In women, 90 urogenital and 439 anorectal CT infections were diagnosed, and 88 urogenital and 96 anorectal NG infections were diagnosed (Fig 2). Among anorectal CT positive MSM, 85.6% (593/693) had rectal-only infections, i.e., without a simultaneous concurrent urogenital CT infection. For women this proportion was 22.1% (97/439) (*p* < 0.001). Among anorectal NG positive MSM, 85.6% (340/397) had rectal-only infections, for women this proportion was 20.8% (20/96) (*p* < 0.001).

### Factors Associated with Anorectal CT and NG

#### MSM

**Fig 3:**
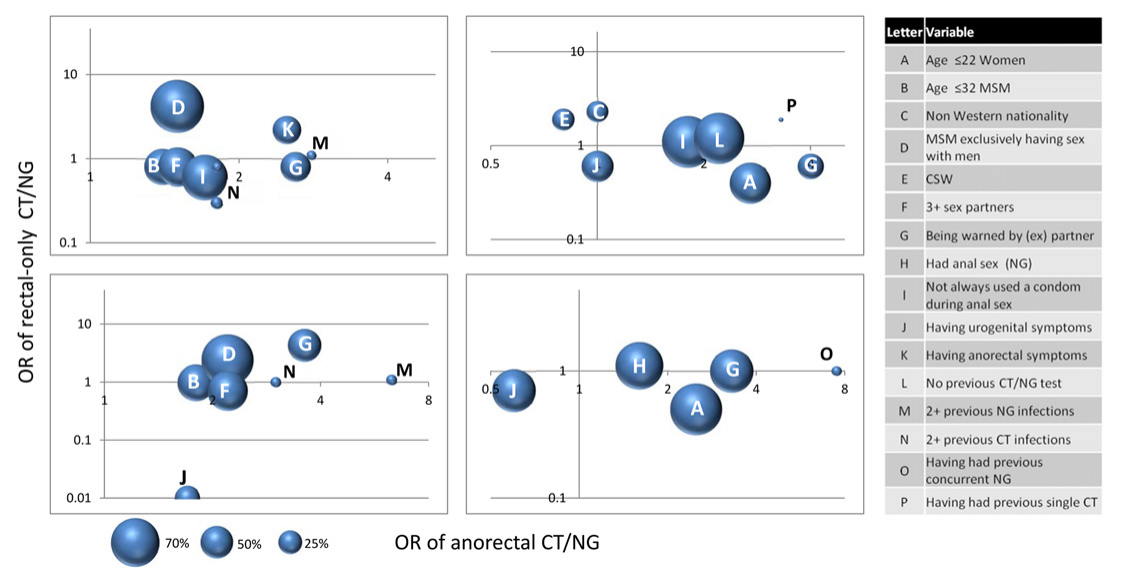
Bubble plot depicting the factors univariably associated with anorectal CT and NG and rectal-only CT and NG in MSM and women including their relative share in the total number of anorectal CT and NG infections. The X-axis represents the odds ratio of anorectal CT or NG, the Y-axis represents the odds ratio of rectal-only CT or NG. The bubble represents the relative share in percentages of anorectal CT and NG infections per associated factor. The variable anal sex was not used for CT to prevent bias by testing indication.

Factors univariately associated with anorectal CT or NG in MSM are presented in S1 Table, independent factors by multivariable analyses are presented in Table 1. We assessed which factors would yield the highest number of CT or NG infections in MSM if targeted in screening by taking into account the absolute number of infections per factor. The largest share of anorectal CT infections was found in MSM exclusively having sex with men, men of a younger age, those reporting having had 3 or more sex partners, who had been warned for STIs by an (ex) partner, or were not always using a condom when practising anal sex (Fig 3). Fig 3 presents the relative share of anorectal infections per factor. For example, screening all MSM who were warned for STIs by an (ex) partner would yield about 50% of all anorectal CT infections in MSM. This factor adds significantly to finding anorectal infections (OR 2.0 on X axis), but does not contribute significantly to finding rectal-only CT (OR 1.0 on Y axis). In total, one or more of the factors presented in Fig 3 was applicable to 98.3% (6973/7094) of MSM. Our data show that using this as a testing algorithm would diagnose 99.3% (n = 691) of anorectal CT infections in MSM.

The largest share of anorectal NG infections was found in MSM who were exclusively having sex with men, who were of a younger age, who had 3 or more sex partners, who had been warned by an (ex) partner, or were experiencing urogenital symptoms (Fig 3). In total, one or more of these factors applied to 97.1% (9261/9534) of MSM. Using this as a testing algorithm would diagnose 99.7% (n = 396) of anorectal NG infections in MSM.

#### Women

Factors univariately associated with anorectal CT or NG in women are presented in S2 Table, independent factors by multivariable analyses are presented in Table 2. We assessed which factors would yield the highest number of CT or NG infections in women if targeted in screening by taking into account the absolute number of infections per factor. The largest share of anorectal CT infections was found in women of a younger age, women who had not visited an STI clinic in the previous 2 years, women who had been warned by an (ex) partner, or those not always using a condom when practising anal sex (Fig 3). In total, 83.9% (3855/4597) of women had one of these factors or a combination thereof. Using this as a testing algorithm would diagnose 93.8% (n = 412) of anorectal NG infections in women. The largest share of anorectal NG infections was found in women of a younger age, those having had anal sex, or women who had been warned by an (ex) partner (Fig 3). In total, 62.8% (6895/10972) of women had one of these factors of a combination thereof. Using this as a testing algorithm would diagnose 85.4% (n = 82) of anorectal NG infections in women.

### Factors Associated with Rectal-Only CT and NG

#### MSM

Factors independently positively associated with rectal-only CT were: exclusively having sex with men, not being a CSW in the past 6 months, and absence of urogenital symptoms. Exclusively having sex with men was the only factor which was also associated with anorectal CT. The largest share of rectal-only CT infections was found in MSM of a younger age, those exclusively having sex with men or MSM who had been warned by an (ex) partner (Table 1 and Fig 3). Factors independently associated with rectal-only NG were: having been warned for STIs by an (ex) partner, absence of an oropharyngeal NG infection, and absence of urogenital symptoms. On the contrary, an oropharyngeal NG infection was associated with anorectal NG infection. Also having been warned for STIs by an (ex) partner was also associated with anorectal NG, however absence of urogenital symptoms was not associated. The largest share of rectal-only NG infections was found in MSM of a younger age, those exclusively having sex with men or those who had been warned by an (ex) partner (Table 1 and Fig 3).

#### Women

Factors independently associated with rectal-only CT infection were: older age (≥28 years), non-Western nationality, and being a CSW. None of these factors were associated with anorectal CT. On the contrary, younger age was associated with anorectal CT. The largest share of rectal-only CT infections were found in older women, women with non Western nationality, women who reported to be a CSW, women who did not always use a condom when practising anal sex, and women who had not visited an STI clinic in the previous 2 years (Table 2 and Fig 3). No factors were independently associated with rectal-only NG infection in our multivariable analyses.

## Discussion

In the present study with 20.662 unique individuals, only a few factors were found to be weakly associated with rectal-only infections, making it difficult to perform selective anorectal screening based on a priori patient characteristics. On the contrary, a wide range of factors was found to be associated with CT and/or NG positivity. Among CT or NG positive MSM, the majority had a rectal-only infection, whereas the majority of women had a concurrent urogenital infection.

We found that the prevalence of anorectal CT in STI outpatient clinic attendees was substantial, at 10%, and comparable for both MSM and women. Anorectal NG prevalence was higher in MSM (4%) as compared to women (1%). The prevalence of anorectal CT and NG in this study is consistent with the prevalence described earlier[15,19,20,23,27]. We found multiple independent associations with anorectal CT and NG infections in MSM and women, which is also consistent with the findings of other studies[8,9,11,12,18–20,23,29,30]. Most previous studies have focused on anorectal CT and NG in MSM; only a few compare MSM and women[13–15,26]. Here, we show that anorectal CT and NG infections are found both in women and MSM attending STI clinics. However, in women, concurrent urogenital and anorectal CT (78%) and NG infections (79%) are detected more frequently than rectal-only infections in contrast to MSM with 14%/ concurrent CT and NG infections. Possible explanations include autoinoculation via vaginal secretions[18,21,23,27], or concurrent transmission during sex.

This study focussed on rectal-only CT and NG infections. It is important to identify rectal-only infections because these remain unnoticed in urogenital site only screening algorithms and, in the case of CT, are possibly sub optimally treated (with single dose azithromycin) when co-occurring with genital CT infection[31,32]. Although the clinical relevance of anorectal infections in women is still largely unknown, it has been suggested that adequate treatment can help limit the spread of CT and NG in the general population[18,19,23], and can reduce susceptibility for HIV infection.

In this study, older age and non-Western nationality were associated with rectal-only infections in women. A Canadian study found older age, and being warned by an (ex)partner for STI to be associated with rectal-only CT infection in women[33]. On the other hand, a US study found young age (<18 years) to be associated with rectal-only CT[15]. This indicates that results are inconclusive and it would be hard to target rectal-only infections in women in practice based on a priori patient characteristics. No associations were found with rectal-only NG in women, as was also the case in a study by Trebach et al.[15] This may be a result of low numbers of rectal-only NG infections in women (n = 20 in our study and n = 50 in the study of Trebach et al.)[15]

In MSM, exclusively having sex with men was associated with both anorectal CT and rectal-only CT. This was the only factor positively associated with rectal-only CT infections in MSM, which makes it hard to target rectal-only infections in MSM in practice based on a priori patient characteristics, as was the case with women. However, encouraging MSM, especially MSM exclusively having sex with men, for anorectal testing yields both anorectal CT and rectal-only anorectal CT. Not having urogenital symptoms and being a CSW were protective for rectal-only CT in this study. A study by Gratrix et al. found that being asymptomatic was associated with a rectal-only CT infection in MSM[34]. However, the absence of urogenital symptoms does not rule out the possibility of a rectal-only CT infection.

Notably, reporting anal sex and anal symptoms were not associated with rectal-only infections in both MSM and women.

When examining data from previous consultations in the past two years in MSM, we found that previous rectal-only CT and NG were associated with anorectal CT. Both previous concurrent and rectal-only NG and previous rectal-only CT infections were associated with anorectal NG. A study by Bernstein et al. reported an increased risk of HIV infection among MSM with rectal infections in the past two years[35]. However, retesting anorectal NG positive MSM would yield only a minority of all anorectal NG infections. Moreover, MSM and women who had a previous rectal-only CT or NG infection were not more likely to have a rectal-only infection.

Selective testing on indication for anorectal CT and NG is currently recommended in the guidelines[5,6] and already widely practised. Previous studies show that anal sex is not associated with anorectal CT in MSM and women, but is associated with anorectal NG[14,20]. Our results indicate that screening all MSM and women who report anal sex or symptoms (selective testing) remains important in order to detect anorectal CT and NG including rectal-only infections. The majority of the study population was tested routinely universally for anorectal NG. In addition to selective testing for anorectal NG, including factors associated with anorectal NG in the testing algorithm could help to detect additional anorectal NG infections in both MSM and women.

Our study has several limitations. A large number of unique individuals were included in this study (N = 20662). However, prevalence of anorectal NG infections in women is low (0.9%) which might have been too low to reach statistical significance when examining associated factors. If individuals had been tested at another care provider in the two years prior to consultation, for example by their general practitioner (GP), this was not taken into account. This could have led to an overestimation of individuals who had not been tested in the two years prior to consultation. However, we presume this bias to be minimal as anorectal testing is limited at GP surgeries[36]. A further limitation of our study is that anorectal test algorithms differed between the two STI clinics. In Amsterdam, all attendees were tested systematically for anorectal NG, but anorectal CT testing was based on reports of anal sex and/or symptoms. In South Limburg, all MSM were routinely tested for anorectal CT and NG and women partially systematically (between May and December 2012), and partially based on report of anal sex and/or symptoms. Such selective testing on indication misses about half of anorectal infections in both MSM[14] and women[20]. It is therefore likely that some anorectal infections were missed using these testing criteria. Possibly, these missed anorectal infections were rectal-only infections, which would bias our results. Moreover, we could have missed factors associated with rectal-only infections since routine universal anorectal screening was missing. Additionally, culture was used for anorectal NG tests in Amsterdam, which might have missed NG infections due to lower sensitivity of culture compared to NAAT. However, the anatomic site distribution of single-site and concurrent CT and NG in MSM and women is comparable with other studies who used routine universal anorectal screening. Moreover, factors associated with anorectal CT and NG found here were comparable with those reported in other studies[8,9,11,12,15,18–20,23,29,30].

In conclusion, prevalence of anorectal CT is substantial in both MSM and women, and prevalence of NG is substantial in MSM. The majority of MSM have rectal-only CT and NG infections, in contrast to women, who are more likely to have concurrent urogenital and anorectal infections. Only a few factors were associated with rectal-only infections, and this makes anorectal screening algorithms based on a priori patient characteristics for MSM and women challenging, because of low discriminatory power. We recommend further research is carried out to inform and optimise anorectal CT and NG case finding.

## Supporting Information

S1 Table Prevalence and factors associated with anorectal chlamydia and gonorrhoea and prevalence and factors associated with rectal-only anorectal chlamydia and gonorrhoea in men who have sex with men by univariable logistic regression. 1.4% (n = 133) of MSM visited a commercial sex worker, of which 6 had anorectal chlamydia and 2 anorectal gonorrhoea. 0.8% (n = 77) of MSM used intravenous drugs of which 7 had anorectal chlamydia and 2 had anorectal gonorrhoea. Both visitation of a commercial sex worker and intravenous drug use were not associated with any of the outcomes, and were not presented in this table due to low numbers. For categorical variables, the reference category is indicated with value ‘1’.
(DOCX)

S2 Table Prevalence and factors associated with anorectal chlamydia and gonorrhoea and prevalence and factors associated with rectal-only anorectal chlamydia and gonorrhoea in women by univariate logistic regression. None of the women were TPHA positive. Fourteen women were HIV positive, one HIV positive woman had anorectal gonorrhoea, this was not tested in analyses due to small numbers. None of the women visited a CSW. For categorical variables, the reference category is indicated with value ‘1’.
(DOCX)

## Abbreviations

MSM: men who have sex with men
CT: *Chlamydia trachomatis*
NG: *Neisseria gonorrhoeae*
OR: Odds ratio
CI: Confidence interval
CSW: commercial sex workers
