# Supplementary material for: Prevalence of and Factors Associated with Rectal-Only Chlamydia and Gonorrhoea in Women and in Men Who Have Sex with Men
Source: PLoS One. 2015 Oct 29;10(10):e0140297. doi: 10.1371/journal.pone.0140297 (PMC4626043; doi:10.1371/journal.pone.0140297)
Supplement: S1 Table — 1.4% (n = 133) of MSM visited a commercial sex worker, of which 6 had anorectal chlamydia and 2 anorectal gonorrhoea. 0.8% (n = 77) of MSM used intravenous drugs of which 7 had anorectal chlamydia and 2 had anorectal gonorrhoea. Both visitation of a commercial sex worker and intravenous drug use were not associated with any of the outcomes, and were not presented in this table due to low numbers. For categorical variables, the reference category is indicated with value ‘1’. (DOCX) [file pone.0140297.s001.docx]

|  |  | Chlamydia | | | | | | | Gonorrhoea | | | | | | |
| --- | --- | --- | --- | --- | --- | --- | --- | --- | --- | --- | --- | --- | --- | --- | --- |
|  |  | Anorectal CT prevalence | | | Con  cur  rent | Rectal-only CT | | | Anorectal NG prevalence | | | Concur  rent | Rectal-only NG | | |
|  | % (n) | % (n) positive | OR | 95% CI | % (n) | % (n) | OR | 95% CI | % (n) positive | OR | 95% CI | % (n) | % (n) | OR | 95% CI |
| Datasource |  |  |  |  |  |  |  |  |  |  |  |  |  |  |  |
| Amsterdam | 87.2 (8327) | 10.1 (593) | 1 |  | 12.6 (75) | 87.4 (518) | 1 |  | 4.3 (356) | 1 |  | 13.8 (49) | 86.2 (307) | 1 |  |
| South Limburg | 12.8 (1222) | 8.2 (100) | 0.8* | 0.6-0.9 | 25.0 (25) | 75.0 (75) | 0.4* | 0.3-0.7 | 3.4 (41) | 0.7 | 0.6-1.1 | 19.5 (8) | 80.5 (33) | 0.7 | 0.3-1.5 |
| Age |  |  |  |  |  |  |  |  |  |  |  |  |  |  |  |
| ≤32 | 37.8 (3612) | 11.1 (298) | 1 |  | 15.4 (46) | 84.6 (252) | 1 |  | 5.3 (191) | 1 |  | 14.7 (28) | 85.3 (163) | 1 |  |
| 33-43 | 30.4 (2901) | 10.0 (224) | 0.9 | 0.8-1.1 | 12.9 (29) | 87.1 (195) | 1.2 | 0.7-2.0 | 4.0 (115) | 0.7* | 0.6-0.9 | 13.9 (16) | 86.1 (99) | 1.1 | 0.6-2.1 |
| ≥44 | 31.8 (3036) | 8.0 (171) | 0.7* | 0.6-0.9 | 14.6 (25) | 85.4 (146) | 1.1 | 0.6-1.8 | 3.0 (91) | 0.5* | 0.4-0.7 | 14.3 (13) | 85.7 (78) | 1.0 | 0.5-2.1 |
| Nationality |  |  |  |  |  |  |  |  |  |  |  |  |  |  |  |
| Western | 85.5(8168) | 9.6 (583) | 1 |  | 14.1 (82) | 85.9 (501) | 1 |  | 4.1 (334) | 1 |  | 15.6 (52) | 84.4 (282) |  |  |
| Non western | 13.2 (1261) | 11.4 (104) | 1.2 | 1.0-1.5 | 17.3 (18) | 82.7 (86) | 0.8 | 0.5-1.4 | 4.9 (62) | 1.2 | 0.9-1.6 | 8.1 (5) | 91.9 (57) | 2.1 | 0.8-5.5 |
| Transmission group |  |  |  |  |  |  |  |  |  |  |  |  |  |  |  |
| MSWM | 86.7 (8283) | 7.0 (53) | 1 |  | 37.7 (20) | 62.3 (33) | 1 |  | 2.1 (26) | 1 |  | 26.9 (7) | 73.1 (19) | 1 |  |
| MSM | 13.3 (1266) | 10.1 (640) | 1.5* | 1.1-2.0 | 12.5 (80) | 87.5 (560) | 4.2* | 2.3-7.8 | 4.5 (371) | 2.2* | 1.5-3.4 | 13.5 (50) | 86.5 (321) | 2.4 | 1.0-5.9 |
| CSW |  |  |  |  |  |  |  |  |  |  |  |  |  |  |  |
| No | 97.3 (9290) | 9.6 (660) | 1 |  | 13.5 (89) | 86.5 (571) | 1 |  | 4.2 (389) | 1 |  | 14.1 (55) | 85.9 (334) | 1 |  |
| Yes | 2.7 (259) | 15.9 (33) | 1.8* | 1.2-2.6 | 33.3 (11) | 66.7 (22) | 0.3* | 0.2-0.7 | 3.1 (8) | 0.7 | 0.4-1.5 | 25.0 (2) | 75.0 (6) | 0.5 | 0.1-2.5 |
| Number sex partners |  |  |  |  |  |  |  |  |  |  |  |  |  |  |  |
| 1 | 24.8 (2365) | 7.5 (128) | 1 |  | 13.3 (17) | 86.7 (111) | 1 |  | 2.5 (60) | 1 |  | 10.0 (6) | 90.0 (54) | 1 |  |
| 2 | 32.3 (3085) | 9.7 (214) | 1.3* | 1.1-1.7 | 12.1 (26) | 87.9 (188) | 1.1 | 0.6-2.1 | 3.6 (112) | 1.5* | 1.1-2.0 | 17.0 (19) | 83.0 (93) | 0.5 | 0.2-1.5 |
| 3+ | 42.6 (4064) | 11.0 (344) | 1.5* | 1.2-1.9 | 15.7 (54) | 84.3 (290) | 0.8 | 0.6-1.5 | 5.5 (222) | 2.2* | 1.7-3.0 | 14.4 (32) | 85.6 (190) | 0.7 | 0.3-1.7 |
| Antibiotics |  |  |  |  |  |  |  |  |  |  |  |  |  |  |  |
| No | 85.7 (8186) | 10.4 (618) | 1 |  | 13.9 (86) | 86.1(532) | 1 |  | 4.3 (350) | 1 |  | 14.0 (49) | 86.0 (301) | 1 |  |
| Yes | 10.4 (991) | 5.9 (44) | 0.5* | 0.4-0.7 | 13.6 (6) | 86.4 (38) | 1.0 | 0.4-2.5 | 3.2 (32) | 0.8 | 0.5-1.1 | 15.6 (5) | 84.4 (27) | 0.9 | 0.3-2.4 |
| Warned |  |  |  |  |  |  |  |  |  |  |  |  |  |  |  |
| No | 80.8 (7716) | 8.2 (461) | 1 |  | 13.4 (62) | 86.6 (399) | 1 |  | 3.0 (234) | 1 |  | 19.7 (46) | 80.3 (188) | 1 |  |
| Yes | 15.6 (1489) | 18.5 (203) | 2.6* | 2.1-3.1 | 15.8 (32) | 84.2 (171) | 0.8 | 0.5-1.3 | 10.0 (149) | 3.6* | 2.9-4.4 | 5.4 (8) | 94.6 (141) | 4.3* | 2.0-9.4 |
| Anal sex |  |  |  |  |  |  |  |  |  |  |  |  |  |  |  |
| No | 12.8 (1220) | 3.5 (13) | 1 |  | 15.4 (2) | 84.6 (11) | 1 |  | 0.9 (11) | 1 |  | 0 (0) | 100 (11) | 1 |  |
| Yes | 83.0 (7921) | 10.3 (650) | 3.2* | 1.8-5.5 | 14.2 (92) | 85.8 (558) | 0.9 | 0.2-4.1 | 4.7 (371) | 5.4* | 3.0-9.9 | 14.6 (54) | 85.4 (317) | 1.0 | 0.5-2.0 |
| Condom always | 58.3 (4621) | 7.6 (188) | 1 |  | 10.1 (19) | 89.9 (169) | 1 |  | 3.4 (111) | 1 |  | 12.6 (14) | 87.4 (97) | 1 |  |
| Condom  not always | 41.3 (3271) | 12.1 (462) | 1.7* | 1.4-2.0 | 15.8 (73) | 84.2 (389) | 0.6 | 0.4-1.0 | 5.6 (260) | 1.7* | 1.4-2.1 | 15.4 (40) | 84.6 (220) | 0.8 | 0.4-1.5 |
| TPHA positive |  |  |  |  |  |  |  |  |  |  |  |  |  |  |  |
| Not tested | 2.4 (227) | 8.8 (20) | 1.0 | 0.6-1.5 | 15.0 (3) | 85.0 (17) | 1.0 | 0.3-3.3 | 3.1 (7) | 0.8 | 0.4-1.6 | 0 (0) | 100 (7) |  |  |
| No | 93.4 (8919) | 9.3 (605) | 1 |  | 14.4 (87) | 85.6 (518) | 1 |  | 4.0 (360) | 1 |  | 14.7 (53) | 85.3 (307) | 1 |  |
| Yes | 4.2 (403) | 20.0 (68) | 2.4* | 1.9-3.2 | 14.7 (10) | 85.3 (58) | 1.0 | 0.5-2.0 | 7.4 (30) | 1.9* | 1.3-2.8 | 13.3 (4) | 86.7 (26) | 1.1 | 0.4-3.3 |
| HIV |  |  |  |  |  |  |  |  |  |  |  |  |  |  |  |
| No | 79.1 (7557) | 8.0 (433) | 1 |  | 15.5 (67) | 84.5 (366) | 1 |  | 3.2 (238) | 1 |  | 16.0 (38) | 84.0 (200) | 1 |  |
| Yes | 2.7 (257) | 23.1 (55) | 3.4* | 2.5-4.7 | 16.4 (9) | 83.6 (46) | 0.9 | 0.4-2.0 | 9.3 (24) | 3.2* | 2.0-4.9 | 16.7 (4) | 83.3 (20) | 0.9 | 0.3-2.9 |
| Unknown | 18.2 (1735) | 14.1 (205) | 1.9* | 1.6-2.2 | 11.7 (24) | 88.3 (181) | 1.4 | 0.8-2.3 | 7.8 (135) | 2.6* | 2.1-3.2 | 11.1 (15) | 88.9 (120) | 1.5 | 0.8-2.9 |
| Urogenital symptoms |  |  |  |  |  |  |  |  |  |  |  |  |  |  |  |
| No | 81.3 (7763) | 9.5 (558) | 1 |  | 10.6 (59) | 89.4 (499) | 1 |  | 3.8 (295) | 1 |  | 1.7 (5) | 98.3 (290) | 1 |  |
| Yes | 15.1 (1442) | 12.1 (106) | 1.3* | 1.1-1.6 | 33.0 (35) | 67.0 (71) | 0.2* | 0.2-0.4 | 6.1 (88) | 1.7* | 1.3-2.1 | 55.7 (49) | 44.3 (39) | 0.01* | 0.01-0.04 |
| Anorectal symptoms |  |  |  |  |  |  |  |  |  |  |  |  |  |  |  |
| No | 91.7 (8756) | 9.2 (578) | 1 |  | 14.1 (83) | 85.6 (495) | 1 |  | 3.6 (318) | na | na | 15.4 (49) | 84.6 (269) | 1 |  |
| Yes | 4.7 (449) | 20.4 (86) | 2.5* | 2.0-3.3 | 12.8 (11) | 87.2 (75) | 1.1 | 0.6-2.2 | 14.6 (65) | na | na | 7.7 (5) | 92.3 (60) | 2.2 | 0.8-5.7 |
| CT urogenital |  |  |  |  |  |  |  |  |  |  |  |  |  |  |  |
| No | 95.4 (9109) | 8.7 (593) | 1 |  | 0.0 (0) | 100 (572) | na | na | 4.0 (363) | 1 |  | 12.4 (45) | 87.6 (318) | 1 |  |
| Yes | 4.3 (412) | 38.2 (100) | 6.5* | 5.0-8.4 | 100.0 (97) | 0.0 (0) | na | na | 8.3 (34) | 2.2* | 1.5-3.1 | 35.3 (12) | 64.7 (22) | 0.3* | 0.1-0.6 |
| CT anorectal |  |  |  |  |  |  |  |  |  |  |  |  |  |  |  |
| Not tested | 25.7 (2455) | na | na | na | na | na | na | na | 0.8 (19) | 0.2* | 0.1-0.3 | 31.6 (6) | 68.4 (13) | 0.3* | 0.1-0.9 |
| No | 67.0 (6398) | na | na | na | na | na | na | na | 4.4 (282) | 1 |  | 13.5 (38) | 86.5 (244) | 1 |  |
| Yes | 7.3 (696) | na | na | na | na | na | na | na | 13.8 (96) | 3.5* | 2.7-4.4 | 13.5 (13) | 86.5 (83) | 1.0 | 0.5-2.0 |
| CT oropharyngeal |  |  |  |  |  |  |  |  |  |  |  |  |  |  |  |
| Not tested | 1.2 (119) | 6.5 (6) | 0.7 | 0.3-1.6 | 16.7 (1) | 83.3 (5) | 0.8 | 0.1-7.0 | 1.7 (2) | 0.4 | 0.1-1.6 | 50.0 (1) | 50.0 (1) | 0.2 | 0.01-2.6 |
| No | 97.4 (9301) | 9.3 (639) | 1 |  | 13.9 (89) | 86.1 (550) | 1 |  | 4.1 (385) | 1 |  | 14.0 (54) | 86.0 (331) | 1 |  |
| Yes | 1.4 (129) | 46.2 (48) | 8.4* | 5.6-12.4 | 20.8 (10) | 79.2(38) | 0.6 | 0.3-1.3 | 7.8 (10) | 1.9* | 1.1-3.7 | 20.0 (2) | 80.0 (8) | 0.7 | 0.1-3.2 |
| NG urogenital |  |  |  |  |  |  |  |  |  |  |  |  |  |  |  |
| No | 97.5 (9308) | 9.7 (674) | 1 |  | 13.8 (93) | 86.2 (581) | 1 |  | 3.7 (40) | 1 |  | 0 (0) | 100 (340) | na | na |
| Yes | 2.5 (234) | 14.1 (19) | 0.7 | 0.4-1.1 | 36.8 (7) | 63.2 (12) | 0.3* | 0.1-0.7 | 24.5 (57) | 8.5* | 6.2-11.7 | 100 (57) | 0 (0) | na | na |
| NG anorectal |  |  |  |  |  |  |  |  |  |  |  |  |  |  |  |
| No | 95.8 (9137) | 8.9 (595) | 1 |  | 13.3 (79) | 86.7 (516) | 1 |  | na | na | na | na | na | na | na |
| Yes | 4.2 (397) | 25.4 (96) | 3.5 | 2.7-4.5 | 21.9 (21) | 78.1 (75) | 0.6* | 0.3-0.9 | na | na | na | na | na | na | na |
| NG oropharyngeal |  |  |  |  |  |  |  |  |  |  |  |  |  |  |  |
| Not tested | 0.5 (50) | 4.2 (1) | 0.4 | 0.1-3.1 | 0 (0) | 100 (1) | na |  | 2.0 (1) | 0.7 | 0.1-5.2 | 100 (1) | 0 (0) | na | na |
| No | 94.4 (9017) | 9.6 (638) | 1 |  | 14.1 (90) | 85.9 (548) | 1 |  | 2.8 (250) | 1 |  | 10.8 (27) | 89.2 (223) | 1 |  |
| Yes | 5.0 (482) | 14.5 (54) | 1.6* | 1.2-2.2 | 18.5 (10) | 81.5 (44) | 0.7 | 0.4-1.5 | 30.4 (146) | 15.3* | 12.1-19.2 | 19.9 (29) | 80.1 (117) | 0.5* | 0.3-0.9 |
| N previous tests |  |  |  |  |  |  |  |  |  |  |  |  |  |  |  |
| 0 | 53.3 (5144) | 10.9 (395) | 1 |  | 13.7 (54) | 86.3 (341) | 1 |  | 4.0 (203) | 1 |  | 13.3 (27) | 86.7 (176) | 1 |  |
| 1-2 | 28.2 (2690) | 10.0 (198) | 0.9 | 0.8-1.1 | 14.6 (29) | 85.4 (169) | 0.9 | 0.6-1.5 | 4.5 (120) | 1.1 | 0.9-1.4 | 17.5 (21) | 82.5 (99) | 0.7 | 0.4-1.4 |
| 3+ | 18.0 (1714) | 6.8 (100) | 0.6* | 0.5-0.8 | 17.0 (17) | 83.0 (83) | 0.8 | 0.4-1.4 | 4.3 (74) | 1.1 | 0.8-1.4 | 12.2 (9) | 87.8 (65) | 1.1 | 0.5-2.5 |
| N previous tests positive CT |  |  |  |  |  |  |  |  |  |  |  |  |  |  |  |
| No previous test | 53.9 (5145) | 10.9 (395) | 1 |  | 13.7 (54) | 86.3 (341) | 1 |  | 4.0 (203) | 1 |  | 13.3 (27) | 86.7 (176) | 1 |  |
| 0 | 38.9 (3711) | 7.3 (204) | 0.6* | 0.5-0.8 | 15.7 (32) | 84.3 (172) | 0.9 | 0.5-1.4 | 3.5 (130) | 0.9 | 0.7-1.1 | 18.5 (24) | 81.5 (106) | 0.7 | 0.4-1.2 |
| 1 | 5.8 (556) | 13.7 (71) | 1.3 | 1.0-1.7 | 15.5 (11) | 84.5 (60) | 0.9 | 0.4-1.8 | 8.8 (49) | 2.4* | 1.7-3.3 | 8.2 (4) | 91.8 (45) | 1.7 | 0.6-5.2 |
| 2+ | 1.4 (137) | 17.7 (23) | 1.8* | 1.1-2.8 | 13.0 (3) | 87.0 (20) | 1.1 | 0.3-3.7 | 10.9 (15) | 3.0* | 1.7-5.2 | 13.3 (2) | 86.7 (13) | 1.0 | 0.2-4.7 |
| N previous tests positive NG |  |  |  |  |  |  |  |  |  |  |  |  |  |  |  |
| No previous tests | 53.9 (5145) | 10.9 (395) | 1 |  | 13.7 (54) | 86.3 (341) | 1 |  | 4.0 (203) | 1 |  | 13.3 (27) | 86.7 (176) | 1 |  |
| 0 | 41.6 (3971) | 7.6 (230) | 0.7* | 0.6-0.8 | 13.9 (32) | 86.1 (198) | 1.0 | 0.6-1.6 | 3.6 (144) | 0.9 | 0.7-1.1 | 15.3 (22) | 84.7 (122) | 0.9 | 0.5-1.6 |
| 1 | 3.7 (355) | 14.9 (49) | 1.4* | 1.1-2.0 | 18.4 (9) | 81.6 (40) | 0.7 | 0.3-1.5 | 9.6 (34) | 2.6* | 1.8-3.8 | 17.6 (6) | 82.4 (28) | 0.7 | 0.3-1.9 |
| 2+ | 0.8 (78) | 25.7 (19) | 2.8* | 1.7-4.8 | 26.3 (5) | 73.7 (14) | 0.4 | 0.2-1.3 | 20.5 (16) | 6.3* | 3.6-11.1 | 12.5 (2) | 87.5 (14) | 1.1 | 0.2-5.0 |
| Previous anorectal CT testing |  |  |  |  |  |  |  |  |  |  |  |  |  |  |  |
| No CT at inclusion | 22.2 (2122) | na | na | na | na | na | na | na | 0.7 (15) | 0.1* | 0.1-0.2 | 26.7 (4) | 73.3 (11) | 0.6 | 0.2-2.2 |
| Never tested before | 41.7 (3985) | 10.5 (419) | 1 |  | 14.8 (62) | 85.2 (357) | 1 |  | 5.2 (207) | 1 |  | 12.6 (26) | 87.4 (181) | 1.6 | 0.9-3.0 |
| Tested negative | 28.8 (2753) | 7.4 (181) | 0.7* | 0.6-0.8 | 13.3 (24) | 86.7 (157) | 1.1 | 0.7-1.9 | 4.0 (111) | 0.8* | 0.6-0.9 | 18.9 (21) | 81.1 (90) | 1 |  |
| Concurrent CT | 0.9 (87) | 16.0 (12) | 1.6 | 0.9-3.0 | 0.0 (0) | 100.0 (12) | na | na | 3.4 (3) | 0.7 | 0.2-2.1 | 0 (0) | 100 (3) | na | na |
| rectal-only CT | 6.3 (602) | 14.2 (81) | 1.4* | 1.1-1.8 | 17.3 (14) | 82.7 (67) | 0.8 | 0.4-1.6 | 10.1 (61) | 2.1* | 1.5-2.8 | 9.8 (6) | 90.2 (55) | 2.1 | 0.8-5.6 |
| Previous anorectal NG testing |  |  |  |  |  |  |  |  |  |  |  |  |  |  |  |
| Never tested | 56.2 (5363) | 10.8 (405) | 1 |  | 14.6 (59) | 85.4 (346) |  |  | 3.9 (209) | 1 |  | 13.9 (29) | 86.1 (180) | 1.1 | 0.6-1.9 |
| Tested negative | 39.2 (3741) | 7.5 (218) | 0.7* | 0.6-0.8 | 12.4 (27) | 87.6 (191) | 1.2 | 0.7-1.7 | 3.7 (138) | 1.0 | 0.8-1.2 | 14.5 (20) | 85.5 (118) | 1 | 0.5-1.8 |
| Concurrent NG | 0.6 (55) | 14.9 (7) | 1.5 | 0.7-3.3 | 14.3 (1) | 85.7 (6) | 1.0 | 0.1-8.5 | 14.5 (8) | 4.2* | 2.0-9.0 | 25.0 (2) | 75.0 (6) | 0.5 | 0.1-2.7 |
| Rectal-only NG | 3.9 (376) | 17.3 (61) | 1.7* | 1.3-2.3 | 21.3 (13) | 78.7 (48) | 0.6 | 0.4-1.5 | 11.2 (42) | 3.1* | 2.2-4.4 | 14.3 (6) | 85.7 (36) | 1.0 | 0.4-2.7 |

S1 Table . Prevalence and factors associated with anorectal chlamydia and gonorrhoea and prevalence and factors associated with rectal-only anorectal chlamydia and gonorrhoea in men who have sex with men by univariable logistic regression. 1.4% (n=133) of MSM visited a commercial sex worker, of which 6 had anorectal chlamydia and 2 anorectal gonorrhoea. 0.8% (n=77) of MSM used intravenous drugs of which 7 had anorectal chlamydia and 2 had anorectal gonorrhoea. Both visitation of a commercial sex worker and intravenous drug use were not associated with any of the outcomes, and were not presented in this table due to low numbers. For categorical variables, the reference category is indicated with value ‘1’.
