# Supplementary material for: Prevalence of and Factors Associated with Rectal-Only Chlamydia and Gonorrhoea in Women and in Men Who Have Sex with Men
Source: PLoS One. 2015 Oct 29;10(10):e0140297. doi: 10.1371/journal.pone.0140297 (PMC4626043; doi:10.1371/journal.pone.0140297)
Supplement: S2 Table — None of the women were TPHA positive. Fourteen women were HIV positive, one HIV positive woman had anorectal gonorrhoea, this was not tested in analyses due to small numbers. None of the women visited a CSW. For categorical variables, the reference category is indicated with value ‘1’. (DOCX) [file pone.0140297.s002.docx]

|  |  | Chlamydia | | | | | | | Gonorrhoea | | | | | | |
| --- | --- | --- | --- | --- | --- | --- | --- | --- | --- | --- | --- | --- | --- | --- | --- |
|  |  | Anorectal CT prevalence | | | Concurrent | Rectal-only CT | | | Anorectal NG prevalence | | | Concurrent | Rectal-only NG | | |
|  |  | % (n) positive | OR | 95% CI | % (n) | % (n) | OR | 95% CI | % (n) positive | OR | 95% CI | % (n) | % (n) | OR | 95% CI |
| Datasource |  |  |  |  |  |  |  |  |  |  |  |  |  |  |  |
| Amsterdam | 89.2 (9913) | 10.0 (339) | 1 |  | 75.2 (255) | 24.8 (84) | 1 |  | 0.9 (87) | 1 |  | 79.3 (69) | 20.7 (18) |  |  |
| South Limburg | 10.8 (1200) | 8.3 (100) | 0.8 | 0.7-1.0 | 87.0 (87) | 13.0 (13) | 0.5* | 0.2-0.9 | 0.8 (9) | 0.9 | 0.4-1.7 | 77.8 (7) | 22.2 (2) | 1.1 | 0.2-5.7 |
| Age |  |  |  |  |  |  |  |  |  |  |  |  |  |  |  |
| ≤22 | 32.1 (3571) | 14.7 (193) | 2.7* | 2.1-3.4 | 82.9 (160) | 17.1 (23) | 1 |  | 1.5 (51) | 2.5* | 1.5-4.2 | 84.3 (43) | 15.7 (8) | 1 |  |
| 23-27 | 34.0 (3782) | 9.3 (132) | 1.6* | 1.2-2.1 | 79.5 (105) | 20.5 (27) | 1.3 | 0.7-2.2 | 0.6 (23) | 1.1 | 0.6-1.9 | 73.9 (17) | 26.1 (6) | 1.9 | 0.6-6.3 |
| ≥28 | 33.8 (3760) | 6.1 (114) | 1 |  | 67.5 (77) | 32.5 (37) | 2.3* | 1.4-4.0 | 0.6 (22) | 1 |  | 72.7 (16) | 27.3 (6) | 2.0 | 0.6-6.7 |
| Nationality |  |  |  |  |  |  |  |  |  |  |  |  |  |  |  |
| Western | 84.8 (9422) | 9.7 (386) | 1 |  | 79.8 (308) | 20.2 (78) | 1 |  | 0.9 (81) | 1 |  | 79.0 (64) | 21.0 (17) | 1 |  |
| Non western | 14.7 (1633) | 9.2 (52) | 1.0 | 0.7-1.3 | 63.5 (33) | 36.5 (19) | 2.3* | 1.2-4.2 | 0.9 (14) | 1.0 | 0.6-1.7 | 85.7 (12) | 14.3 (2) | 0.6 | 0.1-3.1 |
| CSW |  |  |  |  |  |  |  |  |  |  |  |  |  |  |  |
| No | 85.8 (9538) | 9.8 (384) | 1 |  | 79.4 (305) | 20.6 (79) | 1 |  | 0.9 (80) | 1 |  | 80.0 (64) | 20.0 (16) | 1 |  |
| Yes | 14.2 (1575) | 8.2 (55) | 0.8 | 0.6-1.1 | 67.3 (37) | 32.7 (18) | 1.9* | 1.02-3.5 | 1.0 (16) | 1.2 | 0.7-2.1 | 75.0 (12) | 25.0 (4) | 1.3 | 0.4-4.7 |
| Number sex partners |  |  |  |  |  |  |  |  |  |  |  |  |  |  |  |
| 1 | 53.3 (5919) | 10.2 (206) | 1 |  | 79.3 (169) | 20.7 (44) | 1 |  | 0.9 (51) | 1 |  | 76.5 (39) | 23.5 (12) | 1 |  |
| 2 | 26.5 (2941) | 10.1 (125) | 1.0 | 0.8-1.3 | 82.0 (109) | 18.0 (24) | 0.9 | 0.5-1.5 | 0.7 (22) | 0.9 | 0.5-1.4 | 86.4 (19) | 13.6 (3) | 0.5 | 0.1-2.0 |
| 3+ | 20.0 (2219) | 8.0 (85) | 0.8 | 0.6-1.0 | 68.9 (62) | 31.1 (28) | 1.7 | 1.0-3.0 | 1.0 (22) | 1.1 | 0.7-1.9 | 77.3 (17) | 22.7 (5) | 2.0 | 0.3-3.1 |
| Antibiotics |  |  |  |  |  |  |  |  |  |  |  |  |  |  |  |
| No | 82.3 (9144) | 9.8 (348) | 1 |  | 78.0 (276) | 22.0 (78) | 1 |  | 0.9 (85) | 1 |  | 78.8 (67) | 21.2 (18) | 1 |  |
| Yes | 15.5 (1722) | 8.6 (64) | 0.9 | 0.7-1.1 | 78.5 (51) | 21.9 (14) | 1.0 | 0.5-1.9 | 0.5 (9) | 0.6 | 0.3-1.1 | 88.9 (8) | 11.1 (1) | 0.5 | 0.1-4.0 |
| Warned |  |  |  |  |  |  |  |  |  |  |  |  |  |  |  |
| No | 83.1 (9237) | 8.4 (344) | 1 |  | 76.5 (263) | 23.5 (81) | 1 |  | 0.6 (59) | 1 |  | 79.7 (47) | 20.3 (12) | 1 |  |
| Yes | 14.8 (1650) | 26.8 (75) | 4.0* | 3.0-5.3 | 85.3 (64) | 14.7 (11) | 0.6 | 0.3-1.1 | 2.1 (35) | 3.3* | 2.2-5.1 | 80.0 (28) | 20.0 (7) | 1.0 | 0.4-2.8 |
| Anal sex |  |  |  |  |  |  |  |  |  |  |  |  |  |  |  |
| No | 64.4 (7155) | 8.4 (59) | 1 |  | 84.7 (50) | 15.3 (9) | 1 |  | 0.7 (51) | 1 |  | 82.4 (42) | 17.6 (9) | 1 |  |
| Yes | 33.3 (3696) | 9.8 (360) | 1.2 | 0.9-1.6 | 76.9 (277) | 23.1 (83) | 1.2 | 0.7-1.8 | 1.2 (43) | 1.6* | 1.1-2.4 | 76.7 (33) | 23.3 (10) | 1.1 | 0.4-2.8 |
| Condom  Always | 8.4 (312) | 11.2 (33) | 1 |  | 78.8 (26) | 21.2 (7) | 1 |  | 1.3 (4) | 1 |  | 75.0 (3) | 25.0 (1) | 1 |  |
| Condom  Not always | 44.3 (1638) | 18.5 (301) | 1.8* | 1.3-2.6 | 77.1 (232) | 22.9 (69) | 1.1 | 0.5-2.7 | 2.1 (34) | 1.6 | 0.6-4.7 | 79.4 (27) | 20.6 (7) | 0.8 | 0.1-8.7 |
| Urogenital symptoms |  |  |  |  |  |  |  |  |  |  |  |  |  |  |  |
| No | 51.4 (5713) | 9.6 (313) | 1 |  | 77.0 (241) | 23.0 (72) | 1 |  | 1.1 (59) | 1 |  | 78.0 (46) | 22.0 (13) | 1 |  |
| Yes | 46.6 (5174) | 9.5 (106) | 1.0 | 0.8-1.3 | 81.1 (86) | 18.9 (20) | 0.8 | 0.5-1.4 | 0.7 (35) | 0.6* | 0.4-0.97 | 82.0 (29) | 17.1 (6) | 0.7 |  |
| Anorectal symptoms |  |  |  |  |  |  |  |  |  |  |  |  |  |  |  |
| No | 96.1 (10677) | 9.5 (405) | 1 |  | 78.0 (316) | 22.0(89) | 1 |  | 0.9 (91) | 1 |  | 80.2 (73) | 19.8 (18) | 1 |  |
| Yes | 1.9 (210) | 11.1 (14) | 2.5* | 2.0-3.3 | 78.6 (11) | 21.4 (3) | 1.0 | 0.3-3.6 | 1.4 (3) | 1.7 | 0.5-5.3 | 66.7 (2) | 33.3 (1) | 2.0 |  |
| CT urogenital |  |  |  |  |  |  |  |  |  |  |  |  |  |  |  |
| No | 88.2 (9801) | 2.3 (97) |  |  | 0.00 (0) | 100.0 (97) |  |  | 0.6 (54) | 1 |  | 75.9 (41) | 24.1 (13) | 1 |  |
| Yes | 11.7 (1304) | 79.2 (342) | 159.4* | 117.2-216.7 | 100 (342) | 0.0 (0) | na | na | 3.2 (42) | 6.0* | 4.0-9.0 | 83.3 (35) | 16.7 (7) | 0.6 |  |
| CT anorectal |  |  |  |  |  |  |  |  |  |  |  |  |  |  |  |
| Not tested | 58.6 (6516) |  |  |  |  |  |  |  | 0.7 (47) | 0.9 | 0.6-1.4 | 80.9 (38) | 19.1 (9) | 0.6 |  |
| No | 37.4 (4158) |  |  |  |  |  |  |  | 0.8 (32) | 1 |  | 71.9 (23) | 28.1 (9) | 1 |  |
| Yes | 4.0 (439) |  |  |  |  |  |  |  | 3.9 (17) | 5.1* | 2.8-9.3 | 88.2 (15) | 11.8 (2) | 0.3 |  |
| CT oropharyngeal |  |  |  |  |  |  |  |  |  |  |  |  |  |  |  |
| Not tested | 35.1 (3904) | 10.0 (265) | 1.3* | 1.03-1.6 | 77.7 (206) | 22.3 (59) | 0.9 | 0.6-1.5 | 1.0 (39) | 1.4 | 0.9-2.1 | 84.6 (33) | 15.4 (6) | 0.5 |  |
| No | 63.3 (7038) | 8.0 (151) | 1 |  | 76.2 (115) | 23.8 (36) | 1 |  | 0.7 (52) | 1 |  | 73.1 (38) | 26.9 (14) | 1 |  |
| Yes | 1.5 (171) | 56.1 (23) | 14.8* | 7.8-28.0 | 91.3 (21) | 8.7 (2) | 0.3 | 0.1-1.4 | 2.9 (5) | 4.0* | 1.6-10.3 | 100 (5) | 0 (0) | 0 |  |
| NG urogenital |  |  |  |  |  |  |  |  |  |  |  |  |  |  |  |
| No | 97.3 (10817) | 9.5 (417) |  |  | 77.7 (324) | 22.3 (93) |  |  | 0.2 (20) | 1 |  | 0 (0) | 100 (20) | / | / |
| Yes | 1.5 (165) | 29.5 (18) | 4.0* | 2.3-7.0 | 77.8 (14) | 22.2 (4) | 1.0 | 0.3-3.1 | 46.3 (76) | 465.8* | 272.7-795.6 | 100 (76) | 0 (0) | / | / |
| NG anorectal |  |  |  |  |  |  |  |  |  |  |  |  |  |  |  |
| No | 97.9 (10876) | 9.4 (416) | 1 |  | 77.2 (321) | 22.8 (95) | 1 |  | / | / | / | / | / | / | / |
| Yes | 0.9 (96) | 34.7 (17) | 5.1* | 2.8-9.3 | 88.2 (15) | 11.8 (2) | 2.2 | 0.5-9.9 | / | / | / | / | / | / | / |
| NG oropharyngeal |  |  |  |  |  |  |  |  |  |  |  |  |  |  |  |
| Not tested | 35.1 (3897) | 10.0 (265) | 1.2 | 1.0-1.5 | 77.7 (206) | 22.3 (59) | 1.1 | 0.7-1.7 | 1.0 (39) | 2.3* | 1.5-3.7 | 84.6 (33) | 15.4 (6) | 0.6 |  |
| No | 63.9 (7105) | 8.6 (164) | 1 |  | 78.5 (128) | 21.3 (35) | 1 |  | 0.5 (32) | 1 |  | 75.0 (24) | 25.0 (8) | 1 |  |
| Yes | 1.0 (111) | 27.8 (10) | 4.1* | 1.9-8.6 | 77.8 (7) | 30.0 (3) | 1.6 | 0.4-6.4 | 22.5 (25) | 64.2* | 38.5-112.9 | 76.0 (19) | 24.0 (6) | 1.0 |  |
| N previous tests |  |  |  |  |  |  |  |  |  |  |  |  |  |  |  |
| 0 | 67.1 (7455) | 9.5 (292) | 1 |  | 79.1 (231) | 20.9 (61) | 1 |  | 0.8 (58) | 1 |  | 84.5 (49) | 15.5 (9) | 1 |  |
| 1-2 | 26.4 (2937) | 11.3 (130) | 1.2 | 1.0-1.5 | 74.6 (97) | 25.4 (33) | 1.3 | 0.8-2.0 | 1.1 (33) | 1.4 | 0.9-2.2 | 69.7 (23) | 30.3 (10) | 2.4 |  |
| 3+ | 6.5 (721) | 4.6 (17) | 0.5* | 0.3-0.8 | 82.4 (14) | 17.6 (3) | 0.8 | 0.2-2.9 | 0.7 (5) | 0.9 | 0.4-2.2 | 80.0 (4) | 20.0 (1) | 1.4 |  |
| N previous tests positive CT |  |  |  |  |  |  |  |  |  |  |  |  |  |  |  |
| No previous test | 67.1 (7455) | 9.5 (292) | 1 |  | 79.1 (231) | 20.9 (61) | 1 |  | 0.8 (58) | 1 |  | 84.5 (49) | 15.5 (9) |  |  |
| 0 | 31.6 (3510) | 9.3 (131) | 1.0 | 0.8-1.2 | 76.3 (100) | 23.7 (31) | 1.2 | 0.7-1.9 | 1.1 (37) | 1.4 | 0.9-2.0 | 73.0 (27) | 27.0 (10) | 1 |  |
| 1 | 1.2 (129) | 13.4 (13) | 1.5 | 0.8-2.7 | 76.9 (10) | 23.1 (3) | 1.1 | 0.3-4.3 | 0.8 (1) | 1.0 | 0.1-7.1 | 0 (0) | 100 (1) | 2.0 |  |
| 2+ | 0.2 (19) | 16.7 (3) | 1.9 | 0.6-6.6 | 33.3 (1) | 66.7 (2) | 7.6 | 0.7-84.9 | 0 (0) | 0 | 0 | 0 (0) | 0 (0) | E |  |
| N previous tests positive gonorrhoea |  |  |  |  |  |  |  |  |  |  |  |  |  |  |  |
| No previous tests | 67.1 (7455) | 9.5 (292) | 1 |  | 79.1 (231) | 20.9 (61) | 1 |  | 0.8 (58) | 1 |  | 84.5 (49) | 15.5 (9) | 1 |  |
| 0 | 32.4 (3606) | 9.5 (142) | 1.0 | 0.8-1.2 | 76.1 (108) | 23.9 (34) | 1.2 | 0.7-1.9 | 1.0 (36) | 1.3 | 0.8-1.9 | 69.4 (25) | 30.6 (11) | 2.4 |  |
| 1 | 0.4 (48) | 16.7 (5) | 1.9 | 0.7-5.0 | 60.0 (3) | 40.0 (2) | 2.5 | 0.4-15.5 | 4.3 (2) | 5.6 | 1.3-23.6 | 100 (2) | 0 (0) | 1 |  |
| 2+ | 0.01 (4) | (0) | na | na | 0 (0) | 0 (0) | na | na | (0) | na | na | (0) | (0) | na |  |
| Previous anorectal CT testing |  |  |  |  |  |  |  |  |  |  |  |  |  |  |  |
| No CT test at inclusion | 56.5 (6277) | / | / | / | / | / | / |  | 0.7 (42) | 0.6 | 0.4-0.9 | 83.3 (35) | 16.7 (7) | 0.7 |  |
| Never tested before | 35.5 (3950) | 9.9 (390) | 1 |  | 78.5 (306) | 21.5 (84) | 1 |  | 1.1 (43) | 1 |  | 76.7 (33) | 23.3 (10) | 1 |  |
| Tested negative | 6.6 (738) | 6.2 (33) | 0.6* | 0.4-0.9 | 75.8 (25) | 24.2 (8) | 1.2 | 0.5-2.7 | 1.4 (10) | 1.2 | 0.6-2.4 | 80.0 (8) | 20.0 (2) | 0.8 |  |
| Concurrent CT | 0.8 (93) | 11.4 (8) | 1.2 | 0.6-2.5 | 75.0 (6) | 25.0 (2) | 1.2 | 0.2-6.1 | 1.1 (1) | 1.0 | 0.1-7.0 | 0.0 (0) | 100 (1) | na |  |
| Rectal-only CT | 0.5 (55) | 17.8 (8) | 2.0 | 0.9-4.3 | 62.5 (5) | 37.5 (3) | 2.2 | 0.5-9.3 | (0) | na | na | (0) | (0) | na |  |
| Previous anorectal NG testing |  |  |  |  |  |  |  |  |  |  |  |  |  |  |  |
| Never tested before | 82.2 (9140) | 10.1 (367) | 1.7* | 1.1-2.4 | 78.5 (288) | 21.5 (79) | 0.9 | 0.4-2.0 | 0.8 (76) | 1 |  | 81.6 (62) | 18.4 (14) | 1 |  |
| Tested negative | 16.1 (1787) | 8.0 (62) | 1 |  | 74.2 (46) | 25.8 (16) | 1 |  | 1.0 (18) | 1.2 | 0.7-2.0 | 66.7 (12) | 33.3 (6) | 2.2 |  |
| Concurrent NG | 0.3 (35) | 13.6 (3) | 2.0 | 0.9-4.4 | 66.7 (2) | 33.3 (1) | 1.0 | 0.2-6.2 | 5.9 (2) | 7.5* | 1.8-31.7 | 100 (2) | 0 (0) | na |  |
| Rectal-only NG | 0.2 (17) | 16.7 (2) | 3.3* | 1.4-7.6 | 50.0 (1) | 50.0 (1) | 1.9 | 0.4-9.6 | (0) | na | na | (0) | (0) | na |  |

S2 Table. Prevalence and factors associated with anorectal chlamydia and gonorrhoea and prevalence and factors associated with rectal-only anorectal chlamydia and gonorrhoea in women by univariate logistic regression. None of the women were TPHA positive. Fourteen women were HIV positive, one HIV positive woman had anorectal gonorrhoea, this was not tested in analyses due to small numbers. None of the women visited a CSW. For categorical variables, the reference category is indicated with value ‘1’.
